# First person – Chloe Stanton

**DOI:** 10.1242/dmm.049248

**Published:** 2021-09-22

## Abstract

First Person is a series of interviews with the first authors of a selection of papers published in Disease Models & Mechanisms, helping early-career researchers promote themselves alongside their papers. Chloe Stanton is first author on ‘ A mouse model of brittle cornea syndrome caused by mutation in *Zfp469*’, published in DMM. Chloe is a postdoc in the lab of Dr Veronique Vitart at The University of Edinburgh, Edinburgh, UK, investigating the genetic and molecular mechanisms underlying eye diseases.


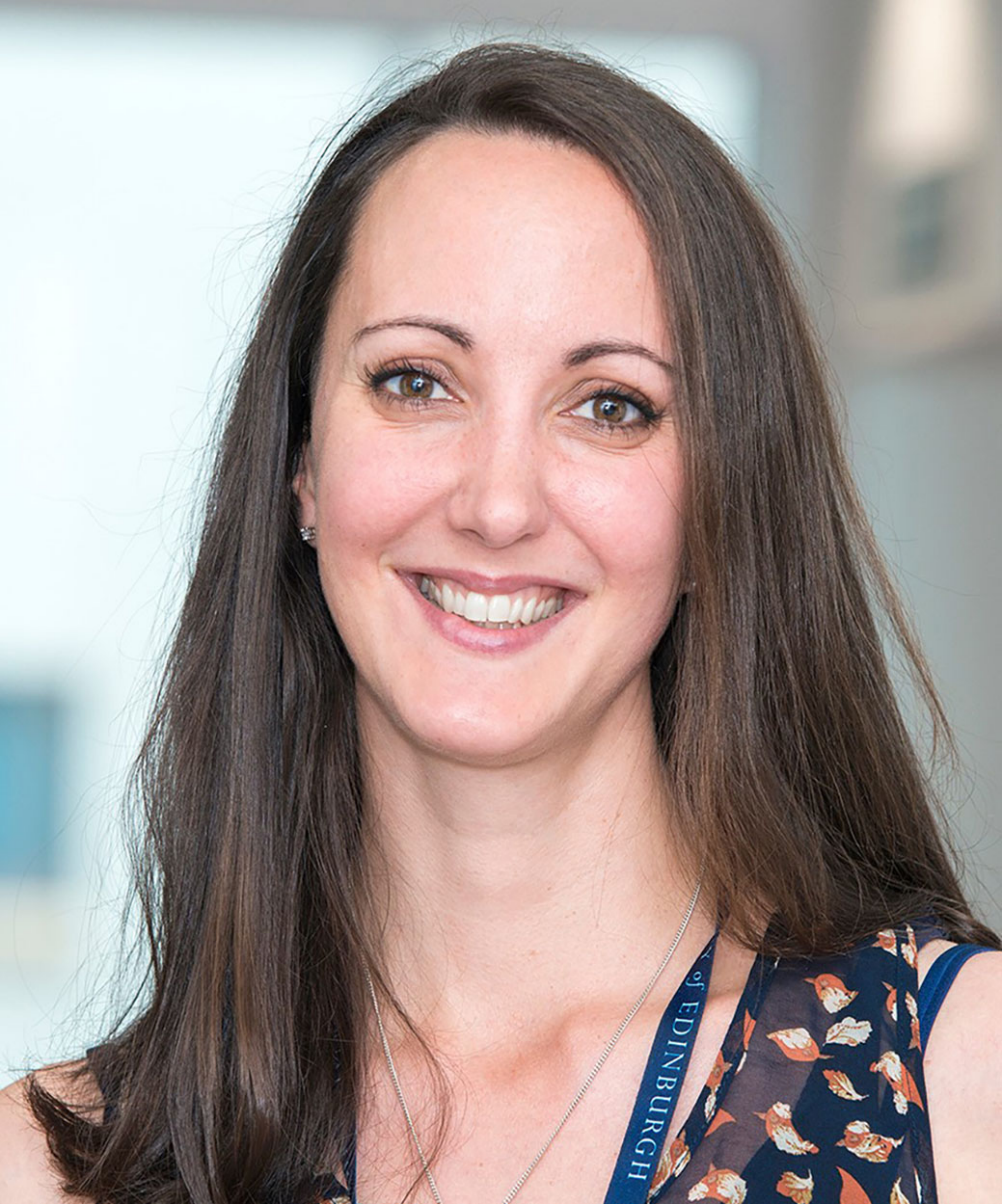


**Chloe Stanton**

**How would you explain the main findings of your paper to non-scientific family and friends?**

Extreme thinning of the cornea – the transparent window at the front of the eye – is seen in the rare genetic condition brittle cornea syndrome (BCS), caused by loss-of-function mutations in the poorly understood gene *ZNF469*. This extreme thinning distorts the cornea and leaves it fragile and prone to rupture, ultimately leading to irreversible blindness in affected individuals. In order to understand more about what ZNF469 does in the cornea, we have made a mouse model of BCS using genome editing to recreate a human disease-causing mutation. Mice with two copies of the mutation have 30% thinner corneas than their siblings without the genetic mutation. Investigating this more closely showed that the thinner corneas arise as a result of thinning of the stroma, normally accounting for 90% of total corneal thickness in humans and 60% in mice. The stroma is composed of strands of connective tissue called collagen fibrils. These fibrils are made by specialised cells called keratocytes, and in our model of BCS the keratocytes make less collagen. This affects the size and number of fibrils formed, altering the thickness and strength of the cornea. We have also made a ‘disease in a dish’ cellular model of dysfunctional keratocytes to let us perform different experiments to determine how and why disrupting *ZNF469* in keratocytes affects the stroma. This may let us investigate potential new treatment options in the future.

**What are the potential implications of these results for your field of research?**

Our mouse model of BCS caused by mutation in *Zfp469* offers important insights into the underlying pathogenic mechanisms of this devastating condition. This disease model also offers a unique entry point for investigating the poorly understood regulatory processes shaping the stroma during development and maintenance of a healthy cornea. This work has important implications for future therapeutic approaches, not only for BCS, but also in other conditions such as keratoconus, where the cornea thins progressively over time. Furthermore, the mechanisms uncovered by this study may have wider implications in connective tissues, given the structural and regulatory functions of collagen type I-rich extracellular matrix across a wide range of tissues, including fat and bone.

“[…] the mechanisms uncovered by this study may have wider implications in connective tissues […]”

**What are the main advantages and drawbacks of the model system you have used as it relates to the disease you are investigating?**

We chose to make a mouse model of BCS to take advantage of two factors – firstly, the development and structure of the mouse corneal stroma is similar to that of human, and secondly, we were able to recapitulate a human disease mutation in the orthologous mouse gene *Zfp469* using CRISPR-Cas9 genome editing. We were also able to use a non-invasive imaging method called anterior-segment optical coherence tomography (AS-OCT) to monitor corneal thickness in our mice over time, from 1 month of age up to 6 months of age. This let us investigate additional features of BCS, including whether the cornea was distorted as is seen in keratoconus or keratoglobus, to detect corneal oedema, and to monitor if corneal thinning was progressive.

None of our mice suffered from corneal rupture, unlike human patients, where corneal rupture normally occurs in early childhood spontaneously or as a result of minor trauma. This may reflect that the mouse cornea has a proportionally thicker epithelial layer than the human cornea, or that the housing conditions of mice offer a more protected environment.

**Figure:**
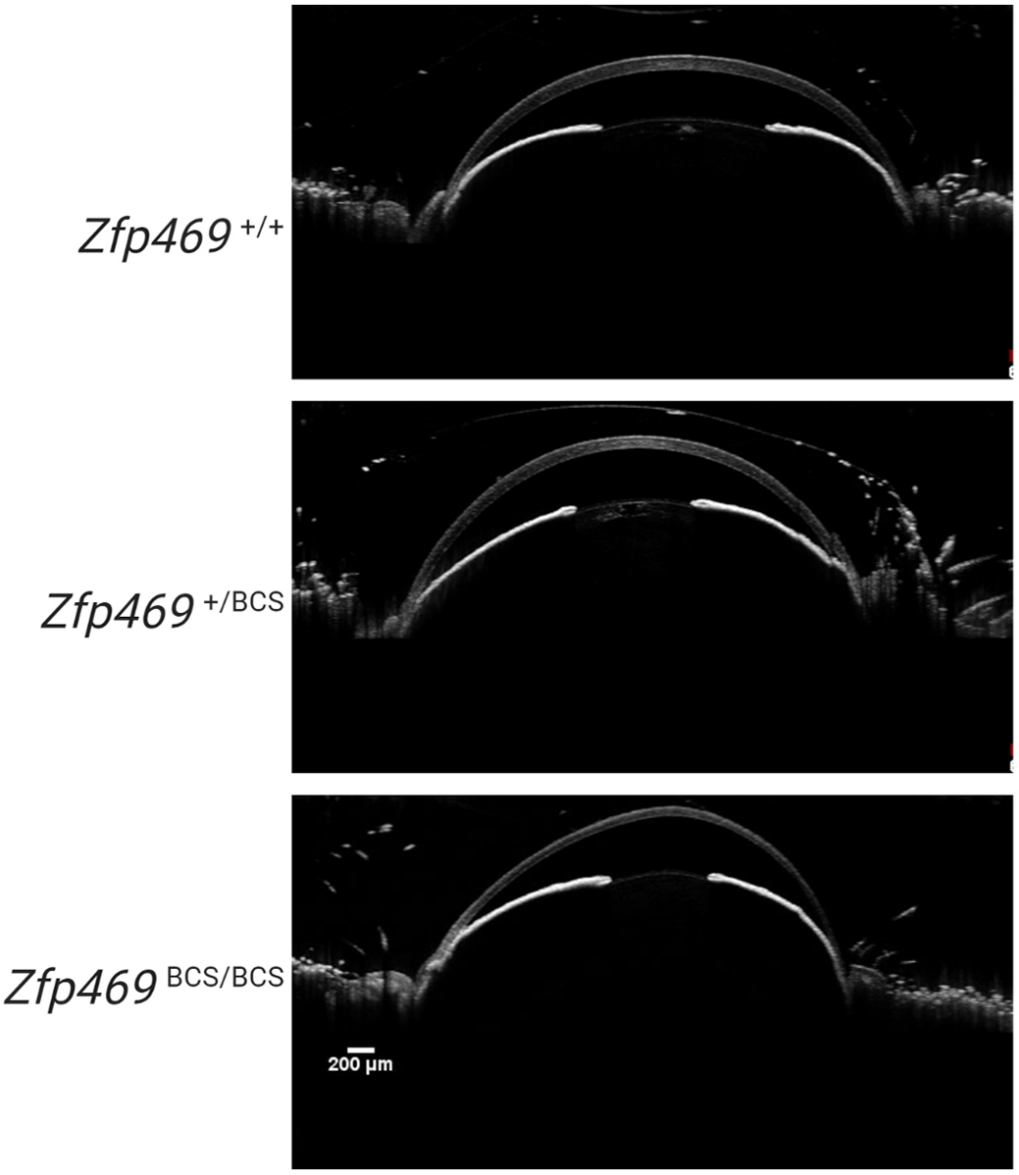
**Corneal distortion is seen by AS-OCT after application of eye drops in *Zfp469*^BCS/BCS^ eyes, but not in heterozygous or wild-type sex-matched littermates, suggesting a loss of biomechanical strength in the extremely thin mutant corneas.** Scale bar: 200 µm.

**What has surprised you the most while conducting your research?**

We investigated the structure of the corneal stroma in our mice. This showed that even though the type I collagen fibrils that formed in the corneal stroma of homozygous mutant mice had a decreased diameter and an increased density, with a resulting lack of biomechanical strength compared to wild types, the lamellae of collagen fibrils retained the exquisite organisation required for corneal transparency.

**Describe what you think is the most significant challenge impacting your research at this time and how will this be addressed over the next 10 years?**

Genome-wide association studies have highlighted that not all genetic variants that alter disease risk are coding variants that result in the expression of a mutated protein with altered function. Deciphering the contribution of regulatory variants to human disease remains challenging, especially when the disease in question affects only a specific cell type, tissue or developmental time point and does not lie in conserved elements of the genome of model organisms. I'm really excited about the recent publication of a single-cell atlas of the different cell types in the human cornea during development using single-cell RNA sequencing (RNA-Seq) and assay for transposase-accessible chromatin with sequencing (ATAC-Seq) to try and provide insight into this issue. I think that this field will continue to develop quickly over the next decade, and, combined with advances in genome editing, we may be able to better model regulatory variants in systems best suited to understanding the disease.

**What's next for you?**

This study showed that our BCS mice had thinner corneas at 1 month of age, at which point the cornea is still developing. My current research – alongside my co-author, Dr Amy Findlay – aims to utilise our model of disease to determine how *Zfp469* contributes to earlier stages of corneal development.
